# Polyamidoamide Dendrimers and Cross-Linking Agents for Stabilized Bioenzymatic Resistant Metal-Free Bovine Collagen

**DOI:** 10.3390/molecules24193611

**Published:** 2019-10-07

**Authors:** Valentina Beghetto, Vanessa Gatto, Silvia Conca, Noemi Bardella, Alberto Scrivanti

**Affiliations:** 1 Department of Molecular Sciences and Nanosystems, University Ca’ Foscari Venice, Via Torino 155, 30172 Mestre (Venice), Italy; vanessa.gatto@unive.it (V.G.); silvia.conca@unive.it (S.C.); noemi.bardella@unive.it (N.B.); scrivant@unive.it (A.S.); 2 Crossing Ltd., Viale della Repubblica 193/b, 31100 Treviso, Italy

**Keywords:** collagen, cross-linking, PAMAM, EDC, enzymatic degradation, DMTMM, dendrimer, tanning

## Abstract

The work reports the use of polyamidoamine dendrimers (PAMAM) and a cross-linking agent, 1-ethyl-3-(3-dimethylaminopropyl)-carbodiimide/*N*-hydroxysuccinimide (EDC/NHS) or 4-(4,6-dimethoxy[1,3,5]triazin-2-yl)-4-methyl-morpholinium chloride (DMTMM), for the thermal stabilization of dermal bovine collagen. The efficiency of EDC/NHS/PAMAM and DMTMM/PAMAM in the cross-linking of collagen is correlated to the increase of the collagen shrinkage temperature (Ts), measured by differential scanning calorimetry (DSC). An alternative enzymatic protocol was adopted to measure the degradability of EDC/NHS/PAMAM tanned hides; these data are correlated to the thermal stability values measured by DSC. In the presence of PAMAMs, EDC/NHS provides very high stabilization of bovine dermal collagen, giving Ts of up to 95 °C, while DMTMM achieves lower stabilization. Preliminary tanning tests carried out in best reaction conditions show that EDC/NHS/PAMAM could be an interesting, environmentally-sustainable tanning system which is completely free of metals, formaldehyde, and phenols. Two new unreported dendrimeric species were synthesized and employed.

## 1. Introduction

Annually, millions of tons of collagen are employed to produce leather goods. The industrial process is based on the valorization of pelts (collagen) produced as waste from food manufacturing, mainly derived from cattle [1]. The major drawbacks encountered in the processing of collagen are its fast biodegradation and low thermal stability, which cause native collagen to degrade rapidly. It is well known that an increment of cross-linking within the protein structure favors its stabilization [2,3,4,5] and enzymatic degradation resistance, which is an indication of enhanced biostability [6,7,8,9]. The nature of these cross-linking bonds depends on the specific chemical agent selected during the tanning process. The main chemical products used industrially are: i) metal complexes, to achieve coordination bonds [10,11,12]; ii) aldehydes, to form covalent bonds [13,14], iii) synthetic or natural tannins, to form ionic bonds, van der Waals interactions, and hydrogen bonds [15,16].

In the tanning industry, basic chromium sulphate is by far the most widely used tanning agent for the stabilization (cross-linking) of hides, as it produces high quality leather (“wet blue”). This process raises considerable environmental concerns due to the high load of chromium in wastewater and solid slurries [17,18,19].

Glutaraldehyde can be adopted for the industrial production of metal free tanned hides or “wet white”. However, the final product has lower chemical-physical characteristics than chrome-tanned leather. Moreover, recent increases in regulatory pressure on aldehydes favors the introduction of more environmentally-sustainable solutions [20,21,22].

**Scheme 1:**
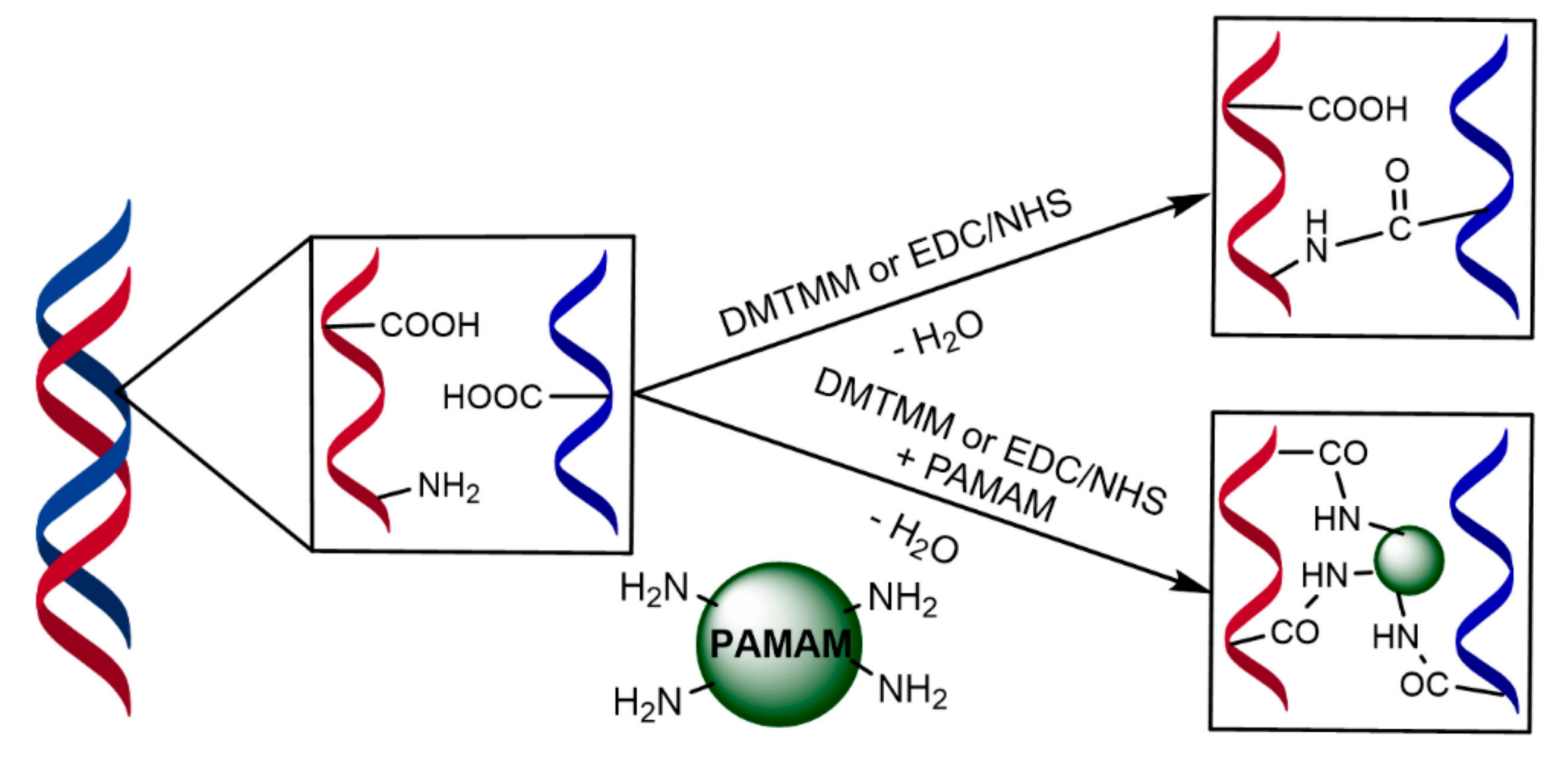
Proposed mechanism of collagen cross-linking with EDC/NHS and DMTMM.

In a recent paper, we reported a study on the economic and environmental sustainability of 4-(4,6-dimethoxy[1,3,5]triazin-2-yl)-4-methyl-morpholinium chloride (DMTMM) as a tanning agent [23]. Similar to DMTMM, EDC/NHS is an active condensing agent for amide bond formation under mild reaction conditions which has been previously employed for the stabilization of collagen for medical and tissue reconstruction [6,7,8,9]. When these chemical agents are employed to stabilize the collagen structure, cross-linked amide bonds are formed between the pendant carboxylic and amine residues present in the protein chains (Scheme 1) [24,25,26,27].

Bovine dermal collagen contains more pendant carboxylic acid groups (COOH_coll_) than amine residues (NH_2Coll_); thus, the degree of collagen stabilization is dependent on less abundant amine functional groups [25]. To overcome this issue, amine-rich dendrimers are sometimes used as coupling auxiliaries to increase the number of free amine groups available [6,7,8,9].

**Figure 1:**
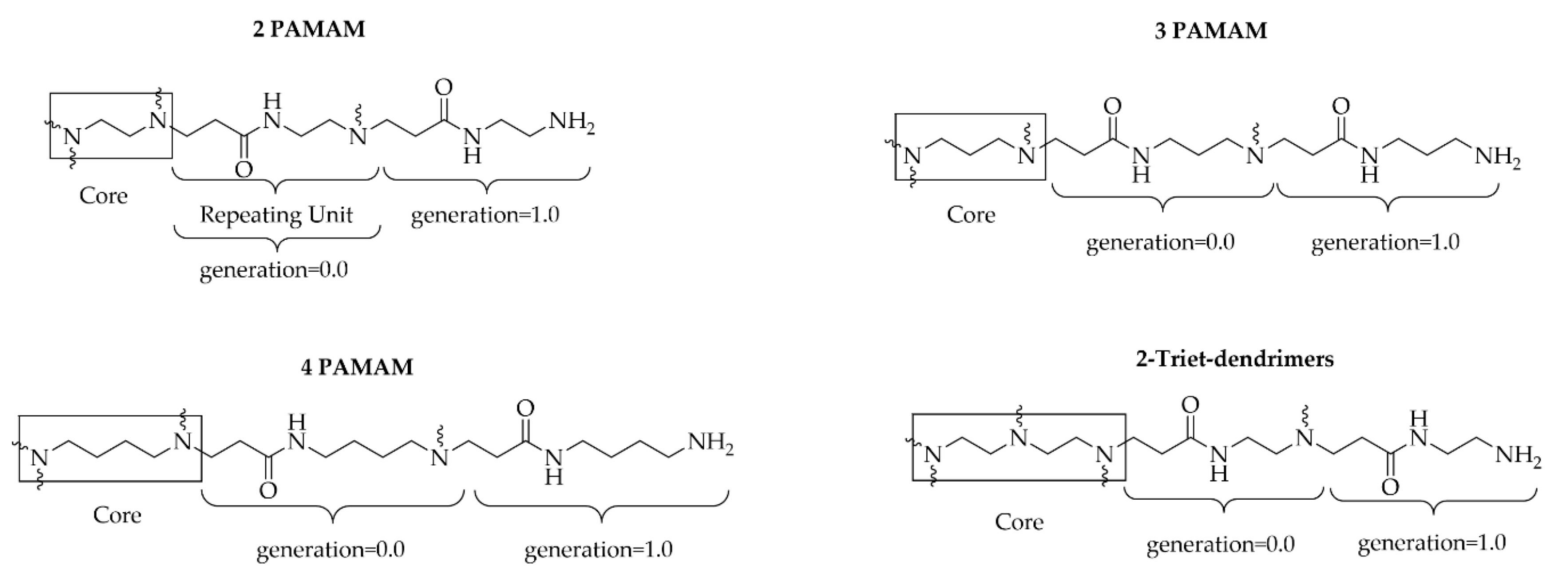
Schematic structure of PAMAM and 2-*Triet*- dendrimers employed in this work.

Tomalia-type polyamidoamine dendrimers (PAMAM) have been successfully used for this purpose in biochemical applications, as they are amine-terminated macromolecules prepared from widely-available feedstock, and are commercially available in the generation from G0.0 to G10.0. The outer shell of PAMAM dendrimers possesses a high number of primary amine end-groups, addressing the deficiency of amine functional groups in collagen reacting with the pendant carboxylic groups present in the collagen protein via amide bond formation (Scheme 1). According to the literature, it is commonly accepted that crossliking agents promote intrahelix interactions. [8,28,29,30]. Preliminary studies carried out employing an amine-rich dendrimer in combination with EDC/NHS and DMTMM gave encouraging results [31]; thus, the aim of this work is to highlight the different activity of EDC/NHS and DMTMM as cross-linking agents in combination with Tomalia-type PAMAM dendrimers (Scheme 1) and their effect on collagen biostability. In Figure 1, the structure of PAMAM dendrimers employed in this work is reported, along with the structure of two new 1,5-diamino-3-azapentane core amine-rich dendrimers (2-*Triet*-G0.0, 2-*Triet*-G1.0) synthesized according to Tomalia’s divergent synthetic methodology [32]. These dendrimers were synthesized in order to gain further data on the reactivity shown by EDC/NHS and DMTMM in the presence of a dendrimer.

Bovine collagen powder was selected as standard substrate for the preliminary cross-linking (or tanning) tests, because it is easy to use, inexpensive, and can be processed with standard laboratory equipment (see experimental section). In this study, this methodology was easy to implement, both in the laboratory and in pilot tests on bovine hides, to produce metal, formaldehyde, and phenol-free leather.

## 2. Results and Discussion

### 2.1. Cross-Linking of Collagen Powder with EDC/NHS or DMTMM in the Presence of a Dendrimer

Preliminary cross-linking experiments were carried out on bovine collagen powder with EDC/NHS and DMTMM without the addition of a dendrimer. Variation in the shrinking temperature (Ts) of collagen was used to evaluate the thermal stability of the cross-linked materials, as previously reported [6,33,34,35]. Since the cross-linking reaction can be influenced by the pH of the medium, a set of experiments was carried out to determine the best pH value for each cross-linking agent. In agreement with the literature, we found that a slightly acidic medium (pH = 5.5) promotes the reaction with EDC/NHS [36,37], while the pH of the tanning bath marginally affected DMTMM efficiency (5.0–9.0 range) [31]. Accordingly, all further tests with DMTMM were carried out at pH 7.0.

As mentioned, these cross-linking agents are responsible for the formation of amide bonds between a carboxylic and an amine functional group (see Figure 1). Princz et al. report that 1.0 g of bovine dermal collagen contains 1.2 mmol of free carboxylic acid groups (COOH_coll_) and 0.8 mmol of free amine groups (NH_2Coll_) [25]. These functional groups will be cross-linked by EDC/NHS or DMTMM to form covalent amide bonds which are dependent on the COOH_coll_/EDC/NHS and COOH_Coll_/DMTMM molar ratios, and influencing the Ts of the treated collagen. 

Moreover, adopting amine-rich auxiliaries, several other variables influence the stabilization of collagen, for instance: the nature and size of the dendrimer, the number of peripherical NH_2_, and the dendrimer to collagen molar ratio [37].

Our studies focused only on the use of low-generation PAMAM dendrimers, i.e., generation 0.0 and 1.0 (having 4 and 8 free NH_2_, respectively), since it has been shown that they avoid the steric hindrance and toxicity issues which are characteristic of higher generations [28,38,39]. In particular, the non-toxicity of the auxiliary is of importance for leather processing (see below).

The Ts data obtained using EDC/NHS in combination with PAMAM dendrimers are presented in Table 1. Cross-linking experiments were carried out according to the protocol optimized in our previous work [31], employing a fixed molar amount of dendrimer, regardless of its generation. Hence, each generation contributes with a different number of free NH_2_ groups, i.e., zero generation with 4 NH_2_ and first generation with 8. The number of NH_2_ present on the outer dendrimer shell is expressed in the fourth column of Table 1, while in the fifth column is specified the molar ratio between COOH_coll_ and the total number of free NH_2_ functional groups calculated as the sum of free NH_2_ available in the collagen protein and those present on the PAMAM dendrimer. It is important to note that using this amount of dendrimers, all experiments were carried out with an excess of -NH_2_ functional groups in order to promote complete exhaustion of -COOH functionalities.

The treatment of collagen powder with EDC/NHS and G0.0s PAMAM implements Ts up to a maximum of 87 °C (compare entries 1–4 of Table 1). The data reported in Table 1 show that the increase in Ts is influenced by the dendrimer molecular weight, since the quantity of NH_2_ on the dendrimer shell is the same. An outstanding enhancement in Ts, up to 95 °C, is obtained when collagen powder is reacted with 2G1.0 PAMAM, which has 8 free NH_2_ groups on the outer surface. It is worth noting that, adversely to G0.0 PAMAMs, when G1.0 dendrimers are employed, collagen stabilization decreases, thereby increasing the molecular weight of PAMAMs. In fact, upon increasing the number of methylene groups in the core of the macromolecule, the Ts of the treated collagen decreases sharply and, eventually, no extra stabilization is observed when employing 4G1.0 PAMAM (compare entry 1 with entry 7 in Table 1).

These results suggest that an increase in the number of free NH_2_ molecules and the molecular weight of the dendrimers leads to an initial increase of Ts, up to a maximum value. The dependence of the Ts value of treated collagen powder versus the molecular weight of the dendrimer employed shows a gaussian trend and a rapid decrease in Ts when higher molecular weight PAMAM are used as auxiliaries

Accordingly, we considered further enhancing cross-linking efficiency by employing auxiliaries with similar molecular weights to 4G0.0 and 4G1.0 PAMAM, with a higher number of NH_2_ molecules on the outer surface. To test this idea, we designed the synthesis of a new family of dendrimeric auxiliaries having 2,2′-diaminodiethylamine as the core molecule. Following a divergent synthetic approach similar to that developed by Tomalia, by recursive reaction of methyl acrylate with 2,2′-diaminodiethylamine, we prepared two new dendritic polyamidoamines, namely 2-*Triet*-G0.0 and 2-*Triet*-G1.0, as depicted in Figure 1 [28]. These new dendrimers have been fully characterized by ESI-MS, ^1^H and ^13^C NMR (see Supplementary Materials).

In agreement with our hypothesis, we found that when 2-*Triet*-G0.0 is employed in combination with EDC/NHS, the collagen Ts value increases up to 90 °C, i.e., a value higher than that obtained with all the other G0.0 PAMAMs tested. On the other hand, with 2-*Triet*-G1.0, no extra stabilization was obtained, even if a higher molar ratio of NH_2_ groups is present. These results agree with the trend reported above in Table 1. With high molecular weight PAMAMs, no extra stabilization is observed, independently of the number of free amine groups available on the PAMAM outer shell.

We eventually investigated collagen stabilization by employing DMTMM in combination with PAMAM dendrimeric auxiliaries (see Table 2). In the first row of Table 2, we have included the data which represent the stabilization achieved with DMTMM alone and a COOH_coll_/DMTMM ratio of 1/2.

Surprisingly, all Ts data reported in Table 2 achieved by treating bovine collagen powder with DMTMM and a PAMAM dendrimer are lower than the stabilization values reached with DMTMM alone [23,31]. In agreement with the literature, we assume that the lower Ts values measured are a consequence of a decrease in the number of cross-linking bonds.

We think that in these reactions, the DMTMM acts as a grafting agent more than a cross-linking one [26,27]. Consequently, in this way, the dendrimers should act as plasticizers and not as cross-linking molecules for collagen, lowering its Ts [5].

### 2.2. Tanning of Bovine Hides with EDC/NHS and PAMAM Dendrimers

Although to the best of our knowledge, no example of a dendrimeric tanning system has been reported in the literature, a few examples are known of hyperbranched polymers employed as tanning agents [6,27]. T. Qiang et al., in their recent work, report the use of a hyperbranched polymer (HPAE-H) in combination with aluminum sulphate as tanning agent affording Ts of 79 °C in best reaction conditions [6]. In this work, aluminum sulfate is required to form bonds between the collagen protein and the hyperbranched polymer. Another example refers to the use of a polyamidoamine as a pre-tanning agent for high-exhaustion chrome tanning [40].

According to the data reported in Table 2, we deemed it interesting to implement the use of EDC/NHS/PAMAM employing 2G1.0 and 2-*Triet*-G0.0 on bovine hides (Table 3). Given that the data reported in Table 2 demonstrate that the efficiency of DMTMM as a tanning agent is depressed in the presence of a dendrimer, no further experiments were carried out with this cross-linking agent.

The tanning experiments were carried out in small drums on freshly delimed hides. Data in Table 3 confirm the high efficacy of the EDC/NHS/PAMAM tertiary system as a tanning agent, reaching Ts values higher than those achieved with most metal-free tanning agents available on the market today (Ts values around 75–78°C) [5]. Moreover, our experiments clearly confirm the correspondence of the Ts data obtained with the two different collagenous substrates (collagen powder and hides) for cross-linking and tanning. This further substantiates the hypothesis that collagen powder may be employed as a simple and economically-viable laboratory standard substrate for preliminary tests of the cross-linking efficiency of tanning agents.

The organoleptic properties of EDC/NHS/PAMAM tanned leather, such as softness, fullness, grain smoothness, flatness, tightness, and general appearance, were evaluated by hand and visual assessment. The characteristics are within the standards required for upper leather samples and have an appreciation value between 6 and 8. An advantage of EDC/NHS/PAMAM is the characteristic white color of the final leather which, compared to chrome tanning, is able to produce brighter colors. Further scaled up experiments will allow us to determine the physical-mechanical properties of EDC/NHS/PAMAM-tanned leather

Compared to metal tanning, the active tertiary cross-linking system reported in this work is able to stabilize collagen devoid of metal salts. Another important advantage of the EDC/NHS/PAMAM system is that only a modest pickling (acidification) with formic acid is required before tanning (about 3.3 wt/wt%), and no NaCl is used. This last feature is very important for the sustainability of the process, since the high concentration of Cl^−^ in tanning wastewater is an increasing environmental concern [41]. The sodium salt is generally required in chrome tanning, as a very low pH is necessary for the penetration of the metal salt inside the skin. In these conditions, swelling of the collagen occurs, which is depressed by the addition of NaCl in concentrations up to 10 wt/wt% of hide processed.

Finally, in contrast to traditional tanning systems, the data reported in the literature clearly show that EDC/NHS acts only as an activating agent, but that it is not retained inside the collagen protein (see Scheme 1) [6,11,12]. Thus, when the EDC/NHS/dendrimer is employed, only the polyamine remains inside the leather. The selection of non-toxic PAMAMs should ensure higher security for the consumer and lower environmental impact of the scraps. In fact, the leather industry has a high environmental impact, also due to the disposal of post-tanning scraps (over 25 wt% of processed hides), that, depending on the tanning system employed, contain heavy metals, formaldehyde, and/or phenols. Scale up studies are in progress to further determine the environmental and economic sustainability of this process.

### 2.3. Enzymatic Degradation Tests

**Figure 2:**
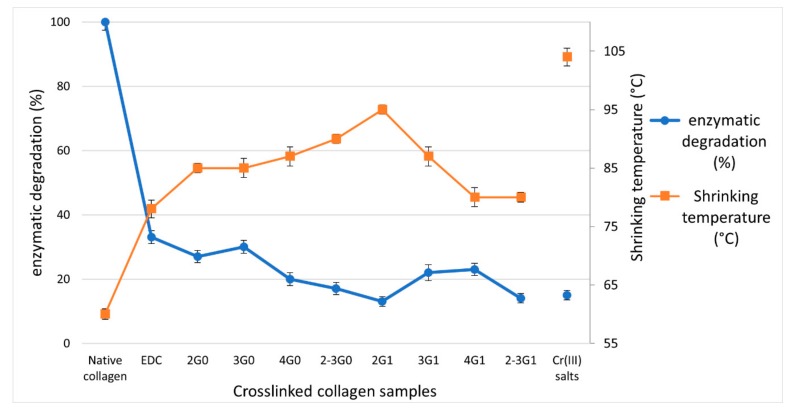
Comparison of Ts (°C) data (measured by DSC) and collagenase wt% degradation of native uncross-linked collagen powder, after cross-linking with COOH_coll_/EDC/NHS 1/2/2 (mol/mol/mol) ratio and cross-linked with chromium sulfate salts (5 wt%).

Enzymatic degradation tests were performed to further examine the crosslinking in the samples and provide information about their biological stability [3,42,43]. The weight loss after collagenase degradation is reported as wt% degradation in Figure 2, and reflects the resistance of the collagen matrices to enzymatic degradation.

The two lines in Figure 2 show an opposite trend which is in good accordance with the fact that when collagen is not cross-linked, or is poorly cross-linked, enzymatic degradation should be high and Ts low; the opposite is true for highly cross-linked samples. In particular, collagen samples which are cross-linked with EDC/NHS (Ts 80 °C) have a loss in weight of 33% after collagenase degradation, while in the best cross-linking conditions employing EDC/NHS and 2-*Triet*-G0.0 (Ts 90 °C) or 2G1.0 (Ts 95 °C) (See Table 2), the degradation wt% decreases to 17 and 15% respectively, i.e., almost equivalent to the value achieved in the presence of Chrome(III) salts. 

It is interesting to note that collagenase degradation tests have been previously adopted to measure collagen stability for pharmaceutical and tissue engineering applications [3,8,43], but very seldom for leather degradation [44,45]. Commonly, degradation tests of tanned leather are carried out by leaving leather samples outdoors in the summer for very long periods (between 150 and 180 days) [46,47]. These methods are very time consuming and, moreover, depend strongly on weather conditions, and are therefore difficult to reproduce. According to the data reported above, enzymatic degradation by collagenase seems to be a very reliable technique to determine leather stability, giving results in very short times, and in good agreement with DSC thermal analysis.

## 3. Materials and Methods 

### 3.1. Materials

Bovine collagen powder was purchased from Filk (Research Institute of Leather and Plastic Sheeting, Freiberg, Germany). First, 2-*Triet*-dendrimers were synthesized according to a literature modification of Tomalia divergent methodology [16]. All other chemicals were purchased from Sigma Aldrich (St. Louis, MO, USA). Delimed bovine pelts were sourced from Santa Croce sull’Arno District (PI, Italy). Differential scanning calorimetry (DSC) of collagen samples was performed on a Netzsch STA 409 cell (Selb, Germany) fitted with an air-cooling compressor at ambient temperature and a controller Netzsch TASC 414/3. The instrument temperature was calibrated using indium as the standard. Collagen samples (about 7.0 mg) were weighed (about 0.1 mg) into aluminum oxidized melting pot and sealed. Samples were heated from 30 °C to 120 °C at a scanning rate of 10 °C/min. A sealed melting pot (about 7.0 mg) filled with Al_2_O_3_ (about 7.0 mg) was used as the reference.

^1^H and ^13^C-NMR spectra were recorded on a Bruker Avance AC 300 spectrometer (Billerica, MA, USA) operating at 300.21 and 75.44 MHz, respectively. ESI-MS analyses were performed using a Finnigan LCQ-Duo ion-trap instrument (San Jose, CA, USA), operating in positive ion mode (sheath gas N_2_, source voltage 4.0 KV, capillary voltage 21 V, capillary temperature 200 °C). Sample solutions were prepared by dissolving the PAMAM dendrimer in methanol. The freshly-prepared solutions were introduced into the ESI source by a syringe pump with 8 mL/min flow rate. 

The shrinking temperature (Ts) of tanned bovine hide was measured according to EN ISO 3380 (IULTCS/IUP 16, 2015). A leather sample was suspended in water, and the rate of heating was maintained at 2 ± 0.2 °C/min. The Ts is the temperature at which the leather shrinks to one-third of its original length. (For more details on the NMR, ESI, and DSC analyses, see the Supplementary Materials).

### 3.2. Methods

#### 3.2.1. Cross-Linking of Bovine Collagen Powder in Combination with PAMAM Dendrimers

First, 300 mg of bovine collagen powder (0.36 mmol COOH_coll_) and 163 mg of 2-*Triet*-G1.0 (0.09 mmol) were dispersed in 30 mL of water under magnetic stirring. After 30 min, 138 mg of EDC (0.72 mmol) and 83 mg of NHS (0.72 mmol) were added to the suspension. After adjusting the pH to 5.5 by the addition of small amounts of 0.1 N NaOH and/or 0.1 N HCl aqueous solutions, stirring was continued for further 4 h; then, the collagen powder was recovered by filtration, air dried, and analyzed by DSC. For DMTMM the same procedure was performed without any pH control.

#### 3.2.2. Tanning of Bovine Hides in the Presence of EDC/NHS and a Dendrimer

First, 100 g of soaked bovine delimed hides (0.12 mol COOH_coll_) dispersed in 150 mL of water were mixed in a drum with 43 g of 2-G1.0 PAMAM (0.03 mol). After adjusting the pH to 5.5 by adding formic acid, 150 mL water solution of EDC (46 g, 0.24 mol) and NHS (28 g, 0.24 mol) were added to the drum. After 18h, the water was discharged, and the hide washed with ammonium sulfate to neutralize the pH.

#### 3.2.3. Organoleptic Tests on Tanned Bovine Hides in the Presence of EDC/NHS and a Dendrimer

Crust leather samples achieved with a conventional syntan retanning protocol were tested for softness, fullness, grain smoothness, grain tightness (break), and general appearance by hand and visual examination. The leathers were rated on a scale of 0–10 for each property by experienced tanners, where higher points indicate better properties.

### 3.3. Enzymatic Hydrolysis

Collagen samples with a dry weight of approximately 5 mg were incubated for 1 h in 0.1M Tris-HCl (pH 7.4) containing 0.04M CaCl_2_ and 0.05 mg/mL NaN_3_ at 37°C. Subsequently, 20 U of bacterial collagenase (Type II from *Clostridium histolyticum*, Sigma: C6885, EC 3.4.24.3) in 1 mL of 0.1M Tris-HCl (pH 7.4) were added. After 2 h at 37°C, the reaction was stopped by the addition of 0.25M EDTA and cooling the mixture on ice. The mixtures were then centrifuged, and the supernatant was separated from the solid residue, which was washed three times with distilled water and subsequently dried. The difference in weight before and after collagenase degradation is reported as wt% degradation. The weights were a mean value of five measurements within an error of ± 2%.

## 4. Conclusions

An extensive study on the activity of EDC/NHS and DMTMM for the cross-linking and tanning of bovine collagen in combination with different PAMAM dendrimers is reported. The Ts of stabilized collagen (measured by DSC) and their biodegradation stability (measured by collagenase hydrolyses) were compared. These data show (see Table 1) that there is a very good correspondence between the cross-linking efficiency of the EDC/NHS/PAMAM tertiary system and the biostability of the treated collagen samples.

The best results were achieved in the presence of the tertiary system EDC/NHS/2G.1, which is a very promising metal-, formaldehyde-, and phenol free cross-linking and tanning agent. This tertiary system achieves very high Ts values (up to 92 °C) compared to commercially-available alternatives, to produce “wet white” leather. The organoleptic characteristics of the tanned leather are comparable with other tanning systems employed industrially for upper leather production.

Moreover, collagen stabilization has been demonstrated to be influenced by PAMAM molecular weight and moles of amine groups present in dendrimer outer shell.

The data reported, moreover, show that Ts values achieved with collagen powder are congruous with those obtained with bovine hides, demonstrating that bovine collagen powder is a valid standard substrate for the preliminary evaluation of the efficacy of tanning agents.

As far as the environmental sustainability of the process is concerned, the use of EDC/NHS/PAMAM lowers the amount of chemicals employed for pickling (NaCl and acids) compared to metal tanning. Another beneficial and environmentally-sustainable feature of the EDC/NHS/PAMAM system is the absence of metals, formaldehyde, or phenols in the wastewater and post-tanning leather scraps. For this reason, this tertiary system can be considered a green tanning system. Scale up studies are in progress to evaluate the physical-mechanical characteristics, Life Cycle Cost, and Life Cycle Analysis of this process.

## Figures and Tables

**Table 1:** Effect of PAMAM auxiliaries on the Ts of bovine collagen powder cross-linked with EDC/NHS.

Entry	Dendrimer	Molecular Weight	PAMAM NH_2_	COOH_coll_/NH_2_^a^ (mol/mol)	Ts (°C)
1	-	-	-	1.2/0.8	80
2	2G0.0	516.4	4	1.2/2.0	85
3	3G0.0	586.8	4	1.2/2.0	85
4	4G0.0	672.0	4	1.2/2.0	87
5	2G1.0	1429.9	8	1.2/3.2	95
6	3G1.0	1612.2	8	1.2/3.2	87
7	4G1.0	1794.6	8	1.2/3.2	80
8	2-*Triet*-G0.0	673.9	5	1.2/2.3	90
9	2-*Triet*-G1.0	1815.3	10	1.2/3.8	80

Reaction Conditions: 300 mg of collagen (0.36 mmol of COOHcoll), 30 mL water, pH 5.5, reaction time: 4h, temperature: 25 °C, COOHcoll/EDC/NHS (mol/mol/mol): 1/2/2. ^a^ For 1g of collagen corresponding to 1.2 mmoles of -COOH and 0.8 mmoles of NH_2_, were added 0.09 mmol of PAMAM dendrimer. The amount of free NH_2_ functional groups is the sum of the peripherical NH_2_ in the dendrimer and the NH_2_ in the collagen protein.

**Table 2:** Effect of PAMAM auxiliaries on the Ts of collagen cross-linked with DMTMM.

Entry	COOH_coll_/DMTMM (mol/mol)	Dendrimer	Surface NH_2_^a^	COOH_coll_/NH_2_^a^ (mol/mol)	Ts (°C)
1	1/2	-	-	1.2/0.8	82
2	1/2	2G0.0	4	1.2/2.0	80
3	1/2	3G0.0	4	1.2/2.0	65
4	1/2	4G0.0	4	1.2/2.0	65
5	1/2	2G1.0	8	1.2/3.2	69
6	1/2	3G1.0	8	1.2/3.2	68
7	1/2	4G1.0	8	1.2/3.2	72
8	1/2	2-*Triet*-G0.0	5	1.2/2.3	76
9	1/2	2-*Triet*-G1.0	10	1.2/3.8	71

Reaction Conditions: 300 mg of collagen (0.36 mmol of COOHcoll), 30 mL water, pH 5.5, reaction time: 4h, temperature: 25 °C, COOHcoll/EDC/NHS (mol/mol/mol): 1/2/2. ^a^ For 1g of collagen corresponding to 1.2 mmoles of -COOH and 0.8 mmoles of NH_2_, were added 0.09 mmol of PAMAM dendrimer. The amount of free NH_2_ functional groups is the sum of the peripherical NH_2_ in the dendrimer and the NH_2_ in the collagen protein.

**Table 3:** Effect of PAMAM auxiliaries on the Ts of bovine hides cross-linked with EDC/NHS.

Entry	Dendrimer	Tanning agent	Surface NH_2_	COOH_coll_/NH_2_^a^ (mol/mol)	Ts (°C)
1	-	EDC/NHS	0	1.2/0.8	79
2	2G1.0	EDC/NHS ^a^	8	1.2/3.2	92
3	2-*Triet*-G0.0	EDC/NHS ^a^	5	1.2/2.3	87

Reaction Conditions: 100 g of bovine hide (0.12 mol of COOHcoll), 300 mL water, pH 5.5, reaction time: 18 h, temperature: 25 °C, COOHcoll/EDC/NHS (mol/mol/mol): 1/2/2, 0.03 mmol of PAMAM dendrimer. ^a^ Sum of free NH_2_ functional groups present in the dendrimer and in the collagen protein.

## Supplementary Materials

Supplementary materials are available online.

## References

[1] Black M., Canova M., Rydin S., Scalet B.M., Roudier S., Sancho D.L. (2013). Best Available Techniques (BAT) Reference Document for the Tanning of Hides and Skins. Industrial Emissions Directive 2010/75/EU.

[2] Chen Y., Dan N., Huang Y., Bai Z., Yang C., Dan W., Cong L. (2019). Functional chemical modification of a porcine acellular dermal matrix with a modified naturally derived polysaccharide crosslinker. J. Appl. Polym. Sci.

[3] Tiong W.H.C., Damodaran G., Naik H., Kelly J.L., Pandit A. (2008). Enhancing Amine Terminals in an Amine-Deprived Collagen Matrix. Langmuir.

[4] Goodarzi H., Jadidi K., Pourmotabed S., Sharifi E., Aghamollaei H. (2019). Preparation and in vitro characterization of cross-linked collagen–gelatin hydrogel using EDC/NHS for corneal tissue engineering applications. Int. J. Biol. Macromol..

[5] Covington A.D. (2009). Tanning Chemistry: The Science of Leather.

[6] Chan J.C.Y., Burugapalli K., Naik H., Kelly J.L., Pandit A. (2008). Amine functionalization of cholecyst-derived extracellular matrix with generation 1 PAMAM dendrimer. Biomacromolecules.

[7] Charulatha V., Rajaram A. (2003). Influence of different crosslinking treatments on the physical properties of collagen membranes. Biomaterials.

[8] Duan X., Sheardown H. (2005). Crosslinking of collagen with dendrimers. J. Biomed. Mater. Res..

[9] Ma L., Gao C., Mao Z., Zhou J., Shen J. (2004). Enhanced biological stability of collagen porous scaffolds by using amino acids as novel cross-linking bridges. Biomaterials.

[10] Bacardit A., van der Burgh S., Armengol J., Ollé L. (2014). Evaluation of a new environment friendly tanning process. J. Clean. Prod..

[11] Ollé L., Jorba M., Font J., Shendrik A., Bacardit A. (2011). Biodegradation of Wet-White Leather. J. Soc. Leather Technol. Chem..

[12] Madhan B., Sundararajan A., Rao J.R., Nair B.U. (2003). Studies on tanning with zirconium oxychloride: Part II development of a versatile tanning system. J. Am. Leather Chem. Assoc..

[13] Han B., Jaurequi J., Bao Wei T., Nimni M.E. (2003). Proanthocyanidin: A natural crosslinking reagent for stabilizing collagen matrices. J. Biomed. Mater. Res..

[14] Khor E. (1997). Methods for the treatment of collagenous tissues for bioprostheses. Biomaterials.

[15] Zengin A.C.A., Crudu M., Maier S.S., Deselnicu V., Albu L., Gulumser G., Bitlisli B.O., Basaran B., Mutlu M.M. (2012). Eco-leather: Chromium-free Leather Production Using Titanium, Oligomeric Melamine-Formaldehyde Resin, and Resorcinol Tanning Agents and the Properties of the Resulting Leathers. Ekoloji.

[16] Marsal A., Cuadros S., Manich A.M., Izquierdo F., Font J. (2017). Reduction of the formaldehyde content in leathers treated with formaldehyde resins by means of plant polyphenols. J. Clean. Prod..

[17] Islam R.M.S., Rahman M.R., Ali M.Y., Uddin M.F. (2018). Environmental Pollution due to Production of Wet-Blue Leather from Goat Skin. IOSR J. Environ. Sci. Toxicol. Food Technol..

[18] Qiang T., Gao X., Ren J., Chen X., Wang X. (2016). A Chrome-Free and Chrome-Less Tanning System Based on the Hyperbranched Polymer. ACS Sustain. Chem. Eng..

[19] Sole R., Taddei L., Franceschi C., Beghetto V. (2019). Efficient Chemo-Enzymatic Transformation of Animal Biomass Waste for Eco-Friendly Leather Production. Molecules.

[20] Huang G.P., Shanmugasundaram S., Masih P., Pandya D., Amara S., Collins G., Arinzeh T.L. (2015). An investigation of common crosslinking agents on the stability of electrospun collagen scaffolds. J. Biomed. Mater. Res. A.

[21] Liu Y., Ma L., Gao C. (2012). Facile fabrication of the glutaraldehyde cross-linked collagen/chitosan porous scaffold for skin tissue engineering. Mater. Sci. Eng. C.

[22] Chen H., Shana Z. (2010). Stabilization of collagen by cross-linking with oxazolidine E-resorcinol. Int. J. Biol. Macromol..

[23] Beghetto V., Agostinis L., Gatto V., Samiolo R., Scrivanti A. (2019). Sustainable use of 4-(4,6-dimethoxy-1,3,5-triazin-2-yl)-4-methylmorpholinium chloride as metal free tanning agent. J. Cleaner Prod..

[24] Taylor M.M., Bumanlag L.P., Brown E.M., Liu C. (2016). Reaction of Protein and Carbohydrates with EDC for Making Unique Biomaterials. J. Am. Leather Chem. Assoc..

[25] Princz M.A., Sheardown H. (2012). Modified Dendrimer Cross-Linked Collagen-Based Matrices. J. Biomater. Sci. Polym. Ed..

[26] Petta D., Eglin D., Grijpma D.W., D’Este M. (2016). Enhancing hyaluronan pseudoplasticity via 4-(4,6-dimethoxy-1,3,5-triazin-2-yl)-4-methylmorpholiniumchloride-mediated conjugation with short alkyl moieties. Carbohydr. Polym..

[27] Pelet J.M., Putnam D. (2011). An In-Depth Analysis of Polymer-Analogous Conjugation using DMTMM. Bioconjugate Chem..

[28] Kaur D., Jain K., Kumar Mehra N., Kesharwani P., Jain N.K. (2016). A review on comparative study of PPI and PAMAM dendrimers. J. Nanopart. Res..

[29] Moon H., Choy S., Park Y., Jung Y.M., Mo Koo Y., Hwang D.S. (2019). Different Molecular Interaction between Collagen and α- or β-Chitin in Mechanically Improved Electrospun Composite. Mar. Drugs.

[30] Collier T.A., Nash A., Birch H.L., de Leeuw N.H. (2018). Effect on the mechanical properties of type I collagen of intra-molecular lysine-arginine derived advanced glycation end-product cross-linking. J. Biomech..

[31] Beghetto V., Agostinis L., Gatto V., Sole R., Zanette D., Conca S., Campana G., Howlett R.J., Setchi R., Cimatti B. (2017). Sustainable carbododiimine and triazine reagents as collagen cross-linking agents in the presence of PAMAM dendrimers. Smart Innovation, Systems and Technologies.

[32] Tomalia D.A., Christensen J.B., Boas U. (2012). Dendrimers, Dendrons, and Dendritic Polymers.

[33] Onem E., Yorgancioglu A., Karavana H.A., Yilmaz O. (2017). Comparison of different tanning agents on the stabilization of collagen via differential scanning calorimetry. J. Therm. Anal. Calorim..

[34] Tang H.R., Covington A.D., Hancock R.A. (2003). Use of DSC to detect the heterogeneity of hydrothermal stability in the polyphenol-treated collagen matrix. J. Agric. Food Chem..

[35] Zhanga Y., Snowb T., Smithb A.J., Holmesa G., Prabakara S. (2019). A guide to high-efficiency chromium (III)-collagen cross-linking: Synchrotron SAXS and DSC study. Int. J. Biol. Macromol..

[36] Yang C. (2012). Enhanced physicochemical properties of collagen by using EDC/NHS-crosslinking. Bull. Mater. Sci..

[37] Gratzer P.F., Lee J.M. (2001). Control of pH Alters the Type of Cross-linking Produced by 1-Ethyl-3-(3-Dimethylaminopropyl)-Carbodiimide (EDC) Treatment of Acellular Matrix Vascular Grafts. J. Biomed. Mater. Res..

[38] Joshi N., Grinstaff M. (2008). Applications of Dendrimers in Tissue Engineering. Curr. Top. Med. Chem..

[39] Alajangi H.K., Natarajan P., Vij M., Ganguli M., Santhiya D. (2016). Role of Unmodified Low Generation – PAMAM Dendrimers in Efficient Non-Toxic Gene Transfection. Chemistry Select..

[40] Ibrahim A.A., Youssef M.S.A., Nashy E.H.A., Eissa M.M. (2013). Using of Hyperbranched Poly(amidoamine) as Pretanning Agent for Leather. Int. J. Polym. Sci..

[41] Gutterres M., Benvenuti J., Fontoura J.T., Ortiz-Monsalve S. (2015). Characterization of Raw Wastewater from Tanneries. J. Soc. Leather Technol. Chem..

[42] Rose J.B., Pacelli S., El Haj A.J., Dua H.S., Hopkinson A., White L.J., Rose F.R.A.J. (2014). Gelatin based materials in ocular tissue engineering. Materials.

[43] Zhong S., Yung L.Y.L. (2008). Enhanced biological stability of collagen with incorporation of PAMAM dendrimer. J. Biomed. Mater. Res. A.

[44] Jackson J.K., Zhao J. (2010). The inhibition of collagenase induced degradation of collagen by the galloyl-containing polyphenols tannic acid, epigallocatechin gallate and epicatechin gallate. J. Mater. Sci. Mater. Med..

[45] Israel-Roming F., Cornea P., Gherghina E., Luta G., Balan D. (2014). Bacterial proteolytic enzymes tested on keratinand collagen based material. Sci. Bullet. Series F. Biotechnol..

[46] Tao-Tao Q., Xiao-Ke C., Xue-Chuan W., Yong-Qiang R., Guo-Xiang Z., Shu-Qin Y. (2011). Biodegradation of chrome-free goat garment leathers. J. Soc. Leath. Tech. Ch..

[47] Bacardit A., Jorba M., Font J., Shendrik A., Ollé L. (2010). Biodegradation of leather tanned with inorganic salts. J. Soc. Leath. Tech. Ch..

