# Supplementary material for: Polyamidoamide Dendrimers and Cross-Linking Agents for Stabilized Bioenzymatic Resistant Metal-Free Bovine Collagen
Source: Molecules. 2019 Oct 7;24(19):3611. doi: 10.3390/molecules24193611 (PMC6803940; doi:10.3390/molecules24193611)
Supplement: Supplementary file 1 [file molecules-24-03611-s001.pdf]

# *Supplementary Materials*

# Polyamidoamide dendrimers and cross-linking agents for stabilized bioenzymatic resistant metal free bovine collagen

*Valentina Beghetto,*†‡ Vanessa Gatto,†‡ Silvia Conca,† Noemi Bardella,† Alberto Scrivanti†*

† Department of Molecular Sciences and Nanosystems, University Ca' Foscari Venice, Via Torino 155, 30172 Mestre (Venice), ITALY

‡ Crossing Ltd., Viale della Repubblica 193/b, 31100 Treviso, ITALY

*Corresponding Author: E-mail: beghetto@unive.it

## Table of contents

Synthesis and characterization of 2-*Triet*- from G-0.5 to G1.0.

**Figure S1.** chemical formula of 2-*Triet*-G-0.5

**Figure S2.** $^{1}H$ NMR spectra of 2-*Triet*-G-0.5

**Figure S3.** $^{13}C$ NMR spectra of 2-*Triet*- G-0.5

**Figure S4.** chemical formula of 2-*Triet*- G0.0

**Figure S5.** chemical formula of 2-*Triet*- G0.5

**Figure S6.** $^{1}H$ NMR spectra of 2-*Triet*-G0.5

**Figure S7.** $^{13}C$ NMR spectra of 2-*Triet*-G0.5

**Figure S8.** chemical formula of 2-*Triet*-G1.0

**Figure S9.** $^{1}H$ NMR spectra of 2-*Triet*-G1.0

**Figure S10.** $^{13}C$ NMR spectra of 2-*Triet*-G1.0

**References**

**Synthesis of 2-*Triet*-G-0.5**

10 mL (9.56 g, 0.11 mol) of methyl acrylate were added drop wise to a solution of diethylentriamine (1.91 g, 0.018 mol) in 100 mL of methanol. The resulting solution was stirred at room temperature for 5 days, then the excesses of methyl acrylate and methanol were evaporated under vacuum to give 2-*Triet*-G-0.5 as a colorless oil (9.58 g, 0.018 mol, 99% yield).

$^{1}$H NMR ($CDCl_3$, 300 MHz) δ: 3.52 (s, 15H), 2.63 (t, 10H, $J_{H,H}$ = 6.9 Hz), 2.36 (s, 8H), 2.29 (t, 10H, $J_{H,H}$ = 6.9 Hz). $^{13}$C{$^{1}$H} NMR ($CDCl_3$, 75 MHz) δ: 172.69 (1C), 172.56 (4C), 52.55 (2C), 51.99 (2C), 51.17 (5C), 50.15 (1C), 49.54 (4C), 32.36 (5C). MS/ESI ($C_{24}H_{43}N_3O_{10}$): m/z 533.29, found: 556.3 $[M+Na]^+$.

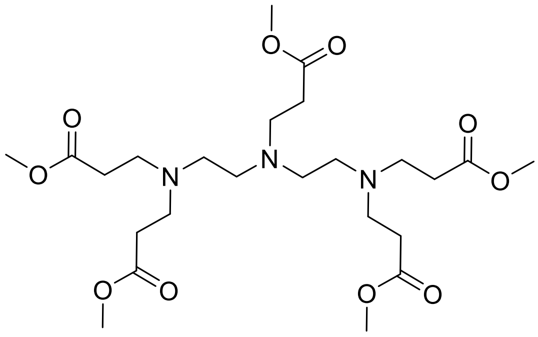


**Figure S1.** chemical formula of 2-*Triet*-G-0.5

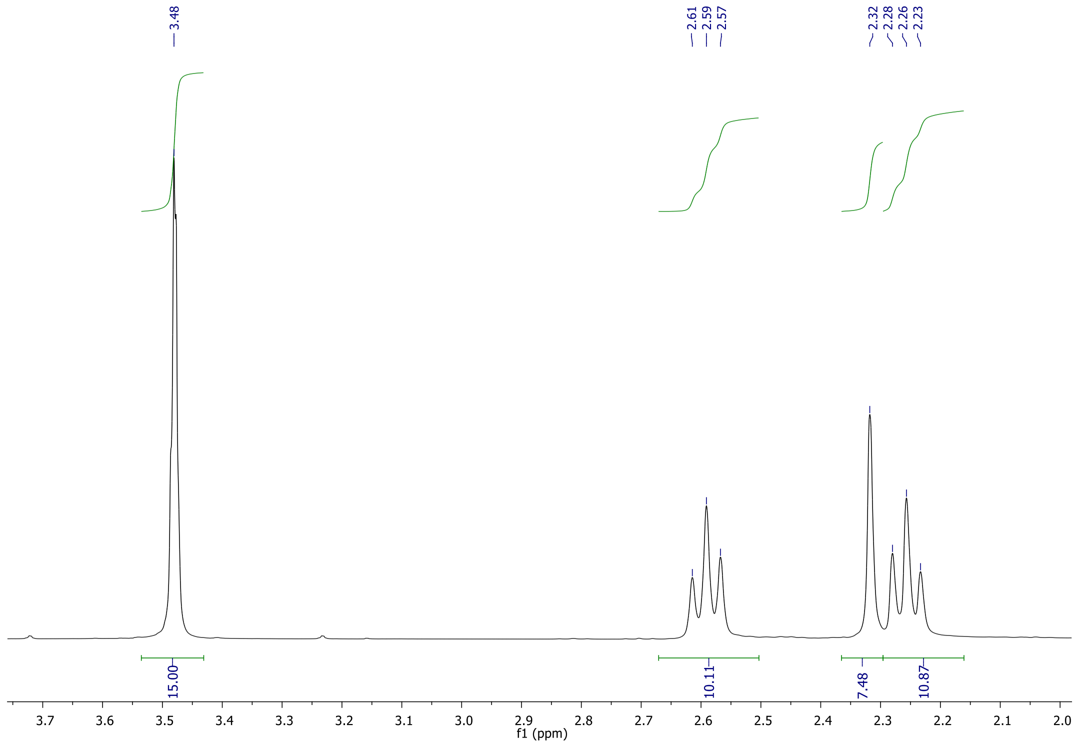


**Figure S2.** $^{1}H$ NMR spectra of 2-*Triet*-G-0.5.

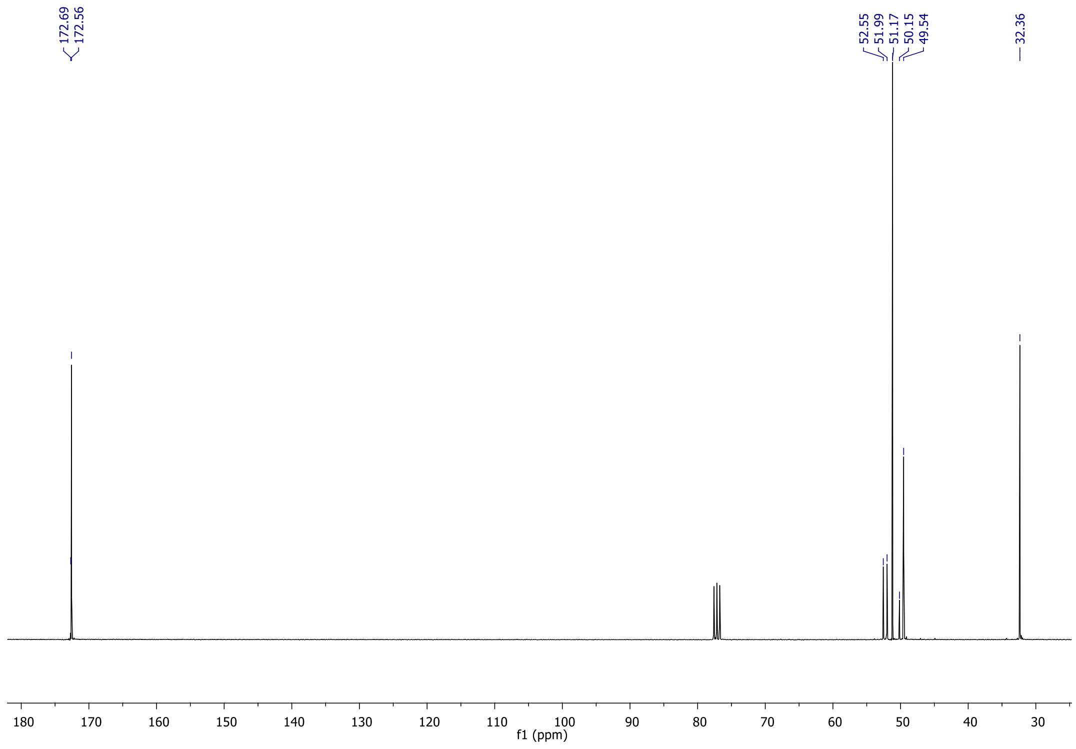


**Figure S3.** $^{13}C$ NMR spectra of 2-*Triet*-G-0.5.

**Synthesis of *2-Triet-G 0.0*** was carried out as reported by Dietrich.[1]

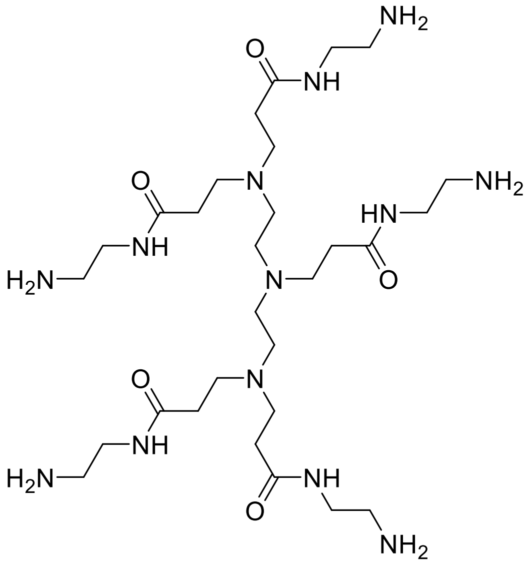


**Figure S4.** chemical formula of 2-*Triet*-G0.0.

**Synthesis of *2-Triet-G 0.5***

2.35 g of methyl acrylate (27.0 mmol) were added drop wise to a methanol solution of 2-*Triet*-G0.0 (0.18 g, 0.27 mmol, in 3 mL). After stirring for 20 h at room temperature, the excesses of methyl acrylate and methanol were removed under high vacuum, to give a colourless oil (0.39 g, 97%).

$^{1}$H NMR ($CDCl_3$, 300 MHz) δ: 7.13 (br, 5H), 3.59 (s, 30H), 3.19 (m, 10H), 2.69 (t, 30H, $J_{H,H}$ = 6.5 Hz), 2.47 (t, 18H, $J$ = 6.5 Hz), 2.39 (t, 20H, $J_{H,H}$ = 6.5 Hz), 2.28 (t, 10H, $J_{H,H}$ = 6.5 Hz). $^{13}$C{1H} NMR ($CDCl_3$, 75 MHz) δ: 172.92 (10C), 172.21 (5C), 52.89 (5C), 51.54 (10C), 50.10 (4C), 49.24 (15C), 37.15 (5C), 33.73 (5C), 32.63 (10C). MS/ESI ($C_{69}H_{123}N_{13}O_{25}$): m/z 1533.87 found 1534.8 $[M+H]^+$, 1556.8 $[M+Na]^+$.

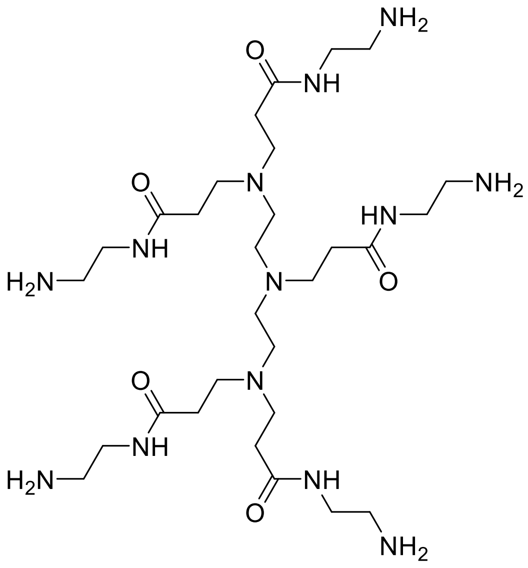


**Figure S5.** chemical formula of 2-*Triet*-G0.5.

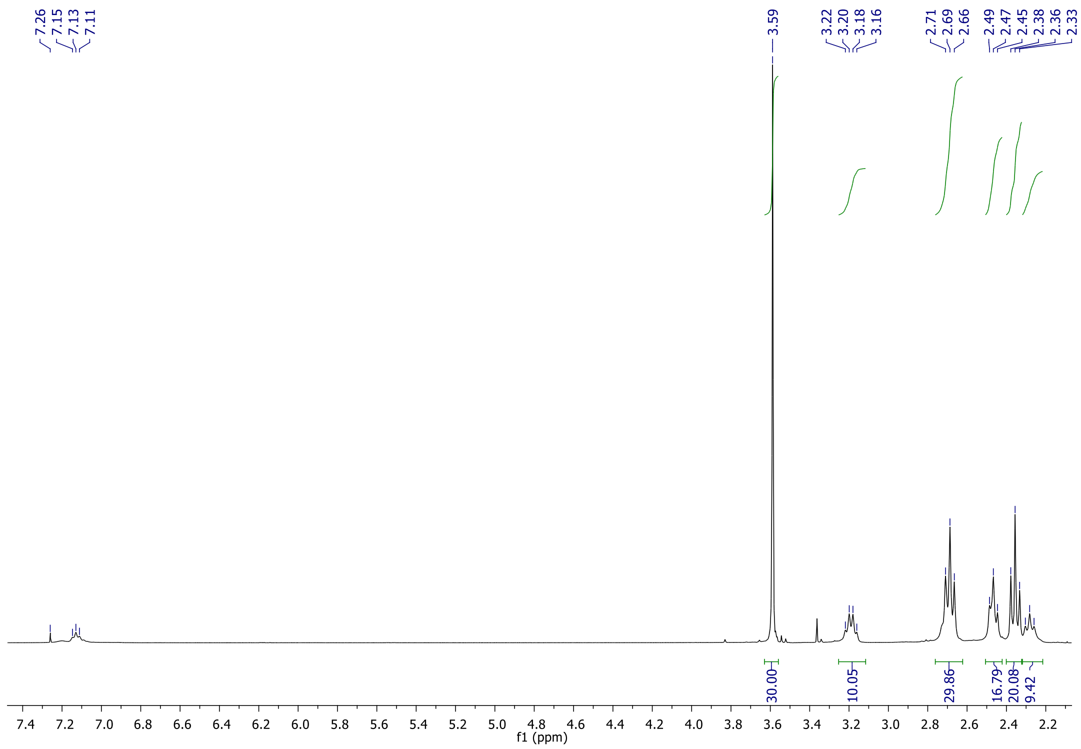


**Figure S6.** ¹H NMR spectra of 2-*Triet*-G0.5.

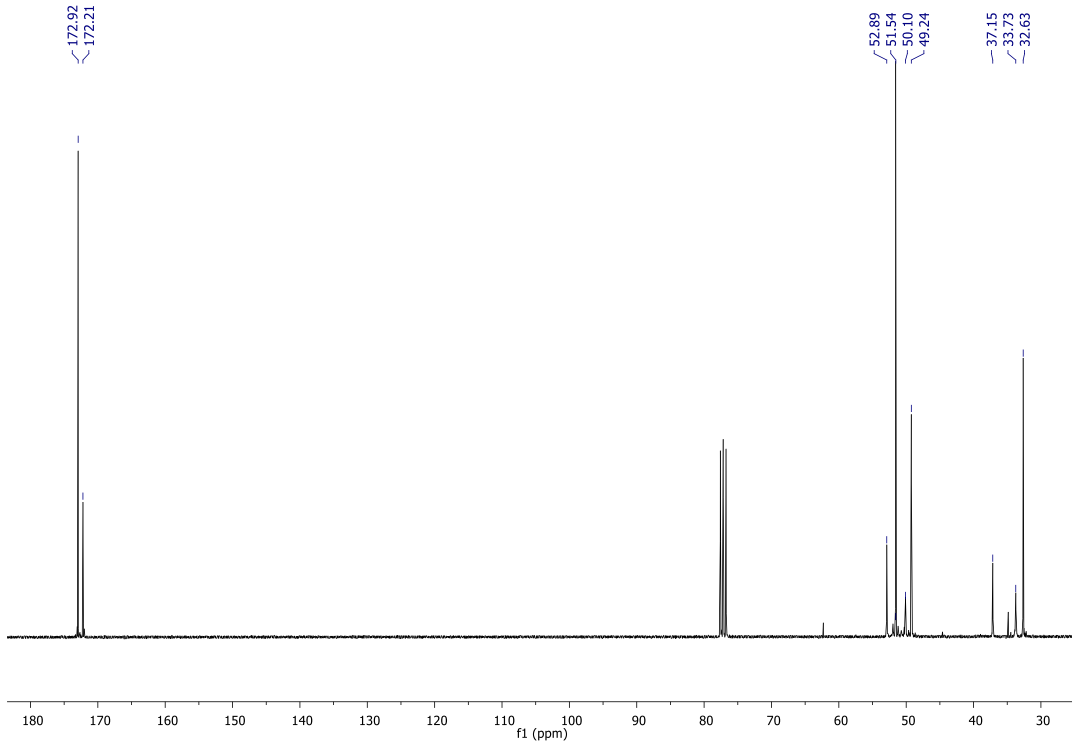


**Figure S7.** $^{13}C$ NMR spectra of 2-*Triet*-G0.5.

**Synthesis of *2-Triet-G1.0***

A methanol solution of ethylenediamine (13 g, 0.21 mol, in 7 mL) was added drop wise to a methanol solution of 2-*Triet*-G0.5 (0.34 g, 0.22 mmol, in 3 mL) at 0 °C. The resulting solution was stirred at room temperature for 5 days, then the excesses of ethylenediamine and methanol were evaporated under vacuum to give a colorless oil (0.39 g, 98%).

$^{1}H$ NMR ($CDCl_3$, 300 MHz) δ: 8.01 (br, 5H), 7.76 (br, 10H), 3.23 (m, 30H), 2.77 (t, 30H, $J_{H,H}$ = 6.9 Hz), 2.71 (bs, 30H), 2.48 (t, 18H, $J_{H,H}$ = 6.9 Hz), 2.32 (t, 30H, $J_{H,H}$ = 6.9 Hz), 1.79 (bs, 20H). $^{13}C\{^{1}H\}$ NMR ($CDCl_3$, 75 MHz) δ: 172.94 (10C), 172.58 (5C), 52.83 (5C) 51.81 (2C), 51.33 (2C), 50.55 (10C), 50.09 (5C), 42.27 (10C), 41.53 (15C), 34.4 (10C), 33.9 (5C). MS/ESI ($C_{79}H_{173}N_{33}O_{15}$): m/z 1814.30 found 1815.3 $[M+H]^+$, 1837.3 $[M+Na]^+$.

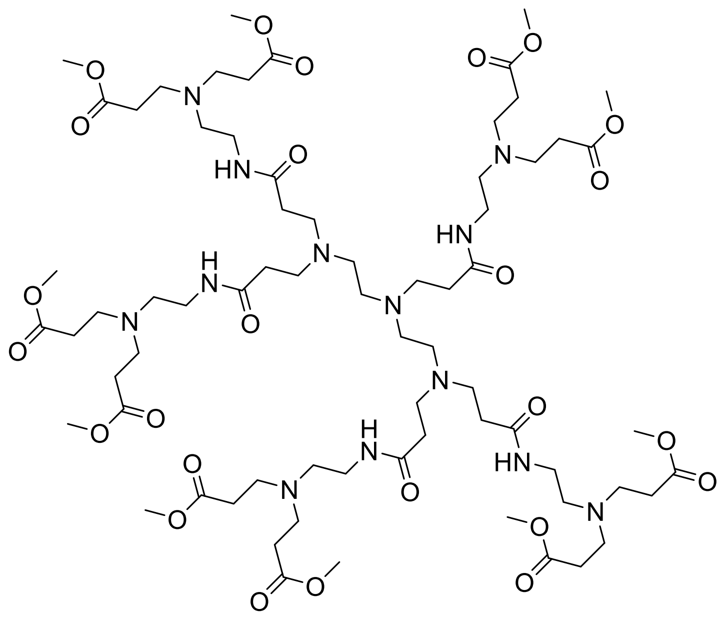


**Figure S8.** chemical formula of 2-*Triet*-G1.0.

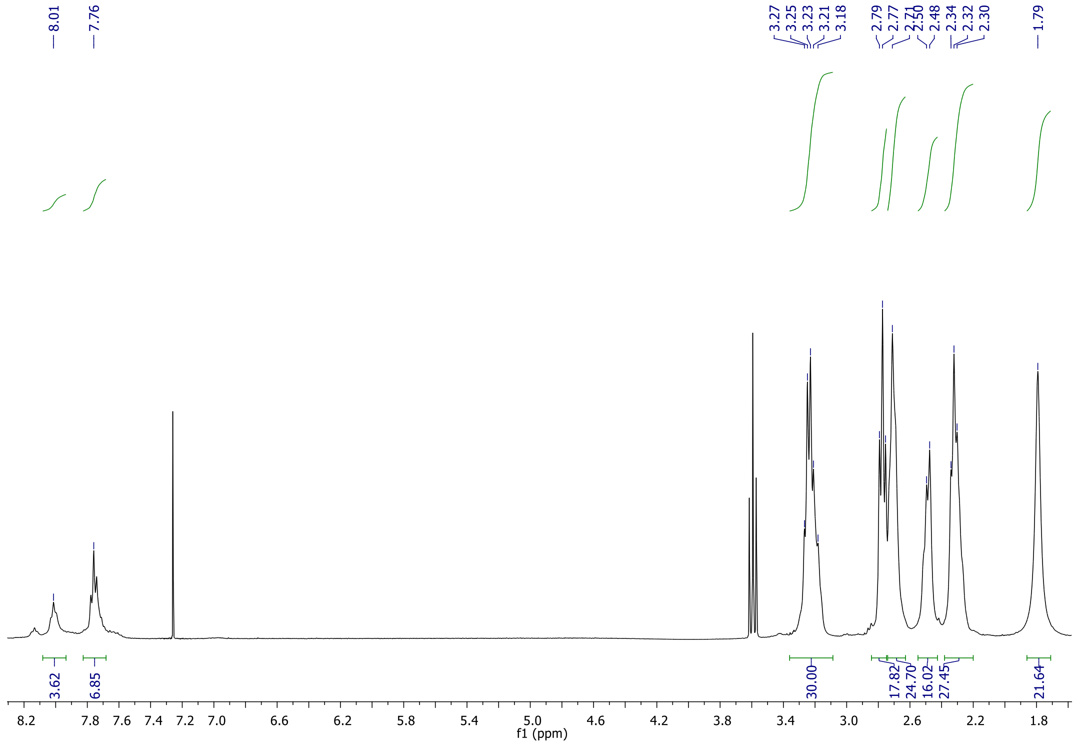


**Figure S9.** $^{1}$H NMR spectra of 2-*Triet*-G1.0.

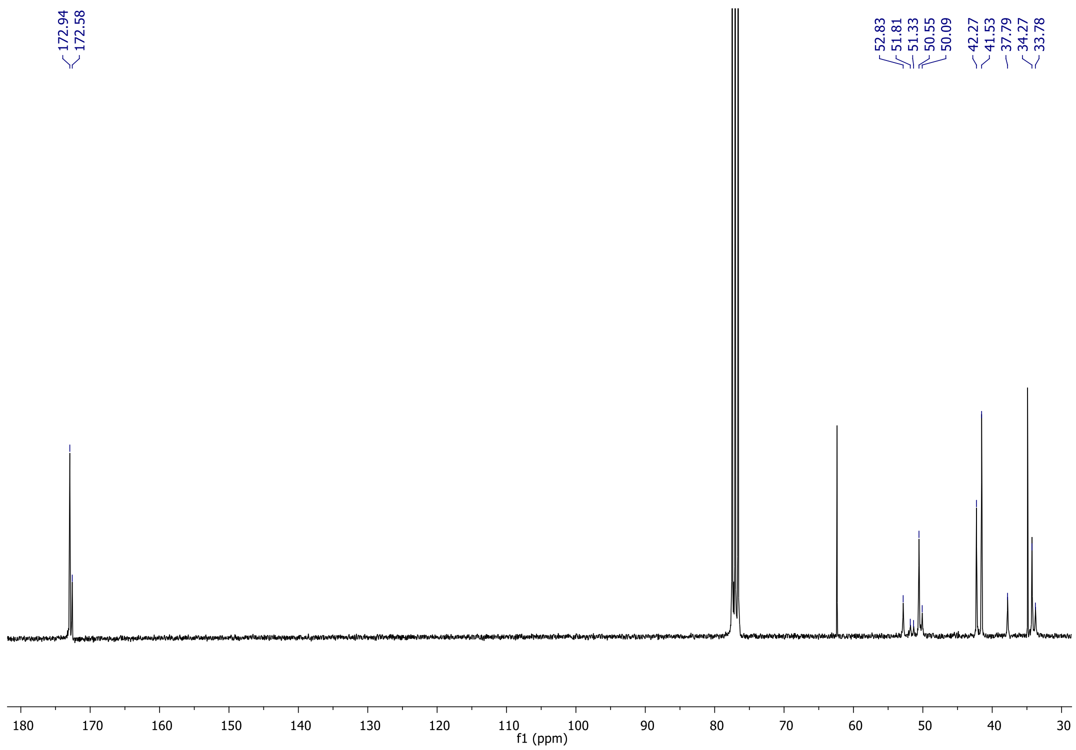


**Figure S10.** $^{13}C$ NMR spectra of 2-*Triet*-G1.0.

## References

(1) Dietrich, S.; Nicolai, A.; Lang, H. Amidoamine-based dendrimers with end-grafted Pd–Fe units: Synthesis, characterization and their use in the Heck reaction. *J. Organomet. Chem.* **2011**, *696*, 739–747.
